# Estimated expenditures for hip fractures using merged healthcare insurance data for individuals aged ≥ 75 years and long-term care insurance claims data in Japan

**DOI:** 10.1007/s11657-018-0448-2

**Published:** 2018-03-30

**Authors:** Takahiro Mori, Nanako Tamiya, Xueying Jin, Boyoung Jeon, Satoru Yoshie, Katsuya Iijima, Tatsuro Ishizaki

**Affiliations:** 1 Research and Development Center for Health Services, Faculty of Medicine, University of Tsukuba, 1-1-1 Tenno-dai, Tsukuba, Ibaraki 305-8575 Japan; 2 Department of General Internal Medicine, Eastern Chiba Medical Center, Togane, Japan; 3 Department of General Medical Science, Graduate School of Medicine, Chiba University, Chiba, Japan; 4 Institute of Gerontology, University of Tokyo, Tokyo, Japan; 5 Human Care Research Team, Tokyo Metropolitan Institute of Gerontology, Tokyo, Japan

**Keywords:** Healthcare expenditure, Long-term care expenditure, Hip fracture, Claims data, Osteoporosis

## Abstract

***Summary*:**

Little is known about hip fracture expenditure in Japan. Using claims data obtained from a core city near Tokyo, we estimated the mean healthcare expenditure and monthly long-term care expenditure post-hip fracture to be ¥2,600,000 (US$29,500) and ¥113,000 (US$1290), respectively.

**Purpose:**

We aimed to estimate healthcare and long-term care expenditures post-hip fracture in Japan.

**Methods:**

Healthcare insurance claims data for adults aged ≥ 75 years were merged with long-term care insurance claims data. We analyzed the data of hip fracture patients who were admitted to non-diagnosis procedure combination/per-diem payment system (DPC/PDPS) hospitals in a core city near Tokyo between April 2012 and September 2013. We estimated healthcare expenditure, namely, the difference between total payments 6 months pre- and 6 months post-hip fracture, and monthly long-term care expenditure for those who did not use long-term care insurance pre-hip fracture, but who commenced long-term care insurance post-hip fracture. We also performed multiple linear regressions to examine the associations of healthcare or long-term care expenditure with various factors.

**Results:**

The estimated mean healthcare (*n* = 78) and monthly long-term care (*n* = 42) expenditures post-hip fracture were ¥2,600,000 (US$29,500) and ¥113,000 (US$1290), respectively. In multiple linear regressions, healthcare expenditure was positively associated with longer duration of hospital stay (*p* = 0.036), and negatively associated with higher Charlson Comorbidity Index scores (*p* = 0.015). Monthly long-term care expenditure was positively associated with higher care-needs level post-hip fracture (*p* = 0.022), and usage of institutional care services (*p* < 0.001).

**Conclusions:**

This is the first study to estimate healthcare and long-term care expenditures post-hip fracture using claims data in Japan. Further studies are needed that include healthcare claims data at both DPC/PDPS and non-DPC/PDPS hospitals to capture the lifelong course of long-term care required post-hip fracture.

## Introduction

The estimated annual incidence of hip fractures in Japan was 175,700 in 2012, which was markedly increased from the incidence of 53,200 in 1987 [1]. A hip fracture not only requires hospitalization and surgery but can also cause a decline in functional status, eventually leading to usage of long-term care. Fractures accounted for 12% of cases in which long-term care insurance was utilized, making them the fourth leading cause in 2013, according to the Comprehensive Survey of Living Conditions that the Japanese Ministry of Health, Labour and Welfare conducts every 3 years [2].

In Japan, the economic burden of hip fractures on society is already assumed to be huge and is expected to further increase as the population ages rapidly. However, little is known about expenditure for hip fractures in Japan. For the past 10 years, only one published study has reported the medical costs of hip fractures based on data from real-world practices in Japanese settings. The study was conducted at three hospitals in Japan, including one DPC/PDPS (Diagnosis Procedure Combination/Per-Diem Payment System) and two non-DPC/PDPS hospitals. The study used the questionnaires sent to the patients or their family members who were admitted for hip fractures, making a lack of generalizability one of the main limitations [3]. In various countries, many studies used claims data to estimate the healthcare expenditure associated with hip fractures, but no such study has been conducted in Japan. The expenditures estimated for other countries may not be applicable to Japan as it has a different healthcare insurance system. Furthermore, to the best of our knowledge, no study has estimated expenditure for long-term care post-hip fracture in Japan using claims data.

The main purpose of our study was to estimate healthcare and long-term care expenditures in elderly patients who experienced a hip fracture in Japan. We also aimed to examine the associations of healthcare or long-term care expenditure with various factors, including age, sex, baseline comorbidity, duration of hospitalization, procedure for a hip fracture, functional status at discharge, and institutional care services post-hip fracture. We hypothesized that longer duration of hospitalization and greater baseline comorbidities were associated with higher healthcare expenditure, and lower levels of functional status at discharge and usage of institutional care services post-hip fracture were associated with higher long-term care expenditure.

## Methods

### Overview of healthcare and long-term care insurance systems and DPC/PDPS in Japan

Japan developed its universal healthcare insurance system in 1961 and a new scheme was implemented in 2008. Every individual aged ≥ 75 years, except for those receiving public assistance, subscribes to the late-stage medical care system for elderly individuals, replacing the healthcare insurance cover for those aged < 75 years [4, 5]. Coverage of the late-stage medical care system for the elderly includes services provided by healthcare professionals, diagnostic tests, prescriptions, surgery, and anesthesia, but it does not cover bed surcharges. The copayment is 10% (or 30% for those with income more than a certain limit) irrespective of the types of services.

The long-term care insurance system was launched in 2000 as a public mandatory insurance system, which was separate from the healthcare insurance system. Those aged ≥ 65 years, as well as those aged between 40 and 64 years with specific aging-related diseases, are eligible for the services, including not only institutional care (e.g., long-term admission or short-term stay to a long-term care facility) but also community- and home-based care (e.g., adult day care, outpatient rehabilitation, home help, or home-visit nursing). Home safety equipment claims are only covered once through long-term care insurance [6, 7]. The copayment is 10% (for those with income more than a certain limit, the copayment was increased to 20% after August 2015) irrespective of the types of services.

In 2003, DPC/PDPS was introduced in Japan, offering a case-mix payment system for acute inpatient care according to diagnoses and procedures. It incorporates two payment systems: a flat-fee per diem payment based on diagnostic categories and a fee-for-service payment based on procedures. In 2012, DPC/PDPS was adopted at more than 1500 hospitals, which cover approximately 53% of the acute care beds [8, 9]. Non-DPC/PDPS hospitals incorporate a fee-for-service payment system only.

### Data source

We obtained healthcare insurance and long-term care insurance claims data between April 2012 and September 2013 from the municipal government of the City of Kashiwa, which is a core city in the Tokyo metropolitan area with a population greater than 400,000 in 2012 (approximately 21% were aged ≥ 65 years as of October 2012). We included only non-DPC/PDPS hospitals in this analysis, as only a small portion of comprehensive DPC/PDPS hospital data (approximately 10%) was seemingly available at the time of this analysis. The datasets obtained contained no personally identifiable information; however, the same dummy ID numbers were assigned to both types of claim datasets. We did not obtain healthcare insurance claims data for those covered by public assistance from the city; therefore, we did not include these in this analysis. The ethics committee of the University of Tsukuba approved this study (Approved number: 1075).

### Participants

**Fig. 1:**
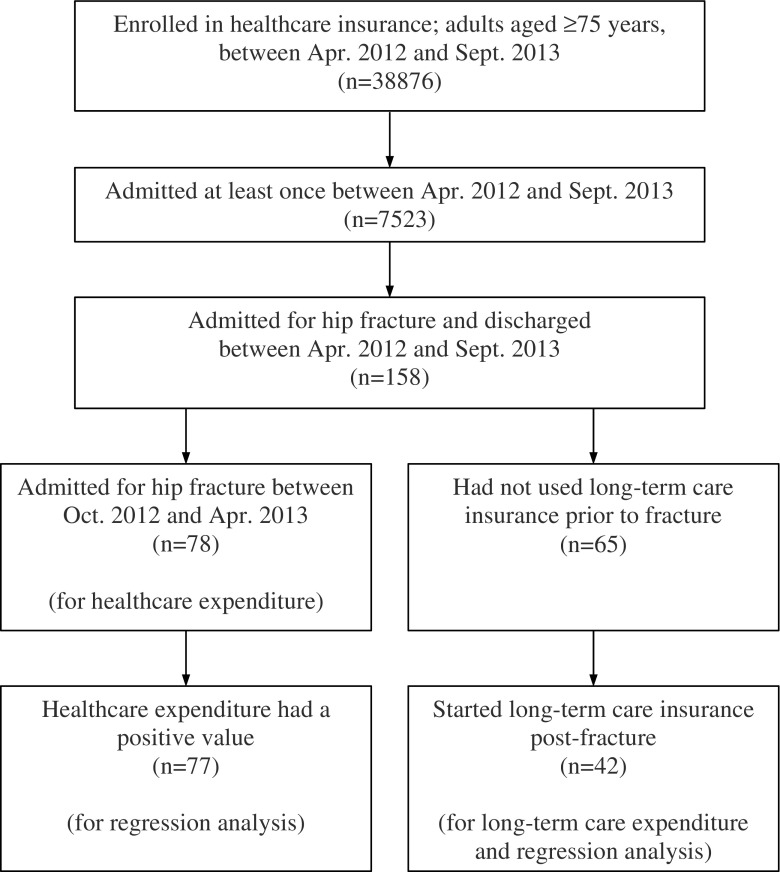
Flow chart of this study

We included those who sustained a hip fracture between April 2012 and September 2013 and were subsequently admitted under the healthcare insurance for the late-stage medical care system for elderly individuals (aged ≥ 75 years). Those who had already been admitted before the beginning of April 2012 or those who were still hospitalized at the end of September 2013 were not included in this analysis (Fig. 1). We focused on elderly individuals aged ≥ 75 years and did not include those aged less than 74 years with healthcare insurance, because the annual incidence rate of hip fractures was much higher for older age groups than that for younger age groups (i.e., the annual incidence rates of hip fracture per 10,000 women were as follows: 8.66, 60–69 years of age; 36.7, 70–79 years of age; 151.0, 80–89 years of age; and 323.3, ≥ 90 years of age) [1]. The International Classification of Diseases, Tenth Revision (ICD-10) codes within the healthcare insurance dataset were used to identify hip fractures. As ICD-10 codes were not helpful in differentiating new hip fractures from previous hip fractures in the dataset, we identified new hip fractures using the procedure codes in the dataset, including hip replacement, open reduction and internal fixation (ORIF), and closed reduction or wiring. Hip fracture patients who were managed non-surgically were excluded as the dataset did not allow us to differentiate new fractures treated non-surgically from old fractures. The ICD-10 codes in this dataset only represented an integer part (e.g., S72 for hip fractures, instead of S72.0); therefore, we also used the disease codes in the dataset, which provided more detailed information such as the anatomical types of hip fractures (e.g., femoral neck, intertrochanteric).

### Measurements

The main purpose of this study was to estimate healthcare and monthly long-term care expenditures for a hip fracture for one person. The expenditures were presented both as Japanese yen (¥) and U.S. dollars (US$) (88 Japanese yen was equivalent to $1 as the mean exchange rate from April 2012 to September 2013), unless specified otherwise [10].

From the healthcare insurance dataset, we obtained data regarding age (provided as the 5-year ranges of birth years [e.g., 1930–1934, 1935–1939]), sex, duration of hospital stays (days), procedure (hip replacement, ORIF, or closed reduction or wiring), anatomical types of hip fractures, and Charlson Comorbidity Index (CCI) scores before or at the time of admission. We used the updated and reweighted version of the CCI scores published in 2011, which are likely to be more suitable for recent administrative data [11]. CCI was originally developed in 1987 and was calculated as the sum of the weighted components such as myocardial infarction, congestive heart failure, peripheral vascular disease, cerebrovascular disease, dementia, chronic pulmonary disease, connective tissue disease, peptic ulcer disease, diabetes mellitus, moderate to severe chronic kidney disease, hemiplegia, leukemia, malignant lymphoma, solid tumor, liver disease, and human immunodeficiency virus/acquired immunodeficiency syndrome [12]. We identified these conditions by the ICD 10 codes [13] and further confirmed the diagnosis by disease codes in the dataset. The conditions with a “suspicious” flag in the claims dataset, which suggested that the diagnoses were placed to justify diagnostic procedures at a hospital with a fee for services system, were not included when we calculated CCI scores. We followed the original definitions of these conditions as much as possible [12].

From the long-term care insurance dataset, we obtained data regarding the duration of usage of long-term care services, usage of institutional care services, and care-needs levels after patients were discharged from a hospital as a proxy of functional status at the time of discharge. Data regarding the duration of long-term care insurance usage for both home-/community-based and institutional care services were provided in terms of months (i.e., if long-term care insurance was used once or more per calendar month, it was considered a 1-month usage of long-term care insurance). Usage of institutional care services in this analysis included not only services under institutional care but also services under home-/community-based services that functioned as institutional care (i.e., a living facility for care for elderly patients with dementia: not for short-term use). The care-needs levels consist of six levels (support-requested level and care-needs levels 1–5), while care level 5 represents the highest level of requirement for long-term care. The long-term care claims dataset did not include medical information (e.g., past medical history).

### Analysis

We merged the claims data of healthcare insurance into that of long-term care insurance at an individual level using dummy ID numbers, which enabled us to add functional status at the time of discharge to the healthcare claims data and to add the diagnosis of a hip fracture and comorbidity to the long-term care claims data.

For analyses of healthcare expenditure, we used incremental payments as a proxy of the expenditure, which were calculated as the difference between the total payments 6 months pre- and 6 months post-hip fracture [14]. The total payment consisted of both national health insurance reimbursement and copayment for the covered services, including both inpatient and outpatient payments. The prescription fees were included, but medication costs were not included in the outpatient payments. We also calculated the difference in expenditures for outpatient care during the period. The claims data were available between April 2012 and September 2013. To ensure that the pre- and post-hip fracture duration (6 months for each) was adequate for this analysis, we included data only for patients who were admitted for hip fractures between October 2012 and April 2013 for obtaining healthcare expenditure. In addition, we calculated attributable payments (directly associated with hip fractures) by including claims representing the ICD 10 codes of only S72 (i.e., hip fractures). We then calculated the proportion of the attributable payments to the incremental payments.

For analyses of long-term care expenditure, we included only those who had not used long-term care insurance before and started using it post-hip fracture. We calculated the long-term care expenditure during the observation period and the duration of usage of long-term care insurance (months), and estimated monthly long-term care expenditure as follows: estimated long-term care expenditure during the observation period divided by the duration of usage of long-term care insurance (months). As a sub-analysis, we also estimated monthly long-term care expenditure, including not only those who started long-term care insurance post-fracture, but also those who did not start long-term care insurance post-fracture. We then performed bootstrapping with 1000 replications to provide bias-corrected confidence intervals of both mean and median healthcare and long-term care expenditures.

Next, we performed multiple linear regressions to examine the associations between healthcare or long-term care expenditure and various factors. For analyses of healthcare expenditure, we included age, sex, duration of stay at a hospital, procedure for hip fracture, comorbidity, and care-needs level at the time of discharge. For analyses of long-term care expenditure, we included age, sex, comorbidity, care-needs level at the time of discharge, and usage of institutional care. We performed regression diagnostics to verify that data met the assumptions underlying linear regressions [15]. Because we were concerned about issues of independence in the healthcare analysis data, we also performed a sensitivity analysis, in which we ran a mixed effect model, treating the hospital variables as a random effect, and other variables as fixed effects [16].

The age variable was divided into two categories (i.e., birth year < 1925–1929 or > 1930–1934, corresponding to age ≥ 86 years or age ≤ 85 years, respectively, as of January 1, 2012). The duration of stay at a hospital (days) was categorized, as follows: (1) ≤ 2 weeks, (2) > 2 weeks, (3) > 4 weeks, (4) > 6 weeks, (5) > 8 weeks, and (6) > 10 weeks. Hip fracture procedures were treated as a categorical variable (hip replacement vs. others) [17]. There was only one case of closed reduction or wiring observed, as shown in the results section below; therefore, this one case was categorized as “others.” The CCI variable was divided into two categories (i.e., CCI index of < 4, ≥ 4) [18]. The care level variable was divided into three categories (i.e., long-term care insurance not required, lower care-needs including support-requested level and care-needs levels 1 and 2, and higher care-needs including care-needs levels 3, 4, and 5) [19] and was treated as an interval variable. The usage of institutional care services was treated as a binary variable.

All statistical tests were performed two-sided (the significant level was set at less than 0.05) using the STATA Version 14.2 (StataCorp LP, College Station, TX, USA).

## Results

Between April 2012 and September 2013, 7523 individuals were admitted to non-DPC/PDPS hospitals at least once in the city under the insurance for the late-stage healthcare system for elderly individuals, and 158 were admitted for a hip fracture and discharged during the period (Fig. 1). Men accounted for 27 fractures (17%) and women for 131 fractures (83%). Ages were presented in 5-year ranges based on the birth year (e.g., 1935–1939). Of the patients, 78% fell into the ranges 1920–1924, 1925–1929, and 1930–1934, corresponding to 87–91 years of age, 82–86 years of age, and 77–81 years of age, as of January 1, 2012, respectively (Table 1). Two individuals out of 158 died during the hospitalization.

**Table 1:** Descriptive characteristics of the study population

	Total (*n* = 158)	Healthcare^a^ (*n* = 78)	Long-term care^b^ (*n* = 42)
Sex			
Men	27 (17%)	15 (19%)	7 (17%)
Women	131 (83%)	63 (81%)	35 (83%)
Birth years (ages as of January 1, 2012)			
1910–1914 (97–101)	2 (1%)	1 (1%)	1 (2%)
1915–1919 (92–96)	19 (12%)	11 (14%)	3 (7%)
1920–1924 (87–91)	39 (25%)	20 (26%)	10 (24%)
1925–1929 (82–86)	44 (28%)	21 (27%)	11 (26%)
1930–1934 (77–81)	41 (26%)	16 (21%)	12 (29%)
1935– (75–76)	13 (8%)	9 (12%)	5 (12%)
Duration of hospital stay (days)			
Mean (SD)	Not applicable^c^	47.4 (23.1)	Not applicable^c^
Median		46	
Procedures and types of fractures^d^			
Hip arthroplasty			
Femoral neck	51	23	17
Other	0	0	0
Open reduction and internal fixation			
Femoral neck	34	17	7
Other	79	42	17
Other procedures (close reduction or wiring)			
Femoral neck	0	0	0
Other	2	1	1
Charlson Comorbidity Index, updated and reweighted version in 2011 at baseline^e^			
Mean (SD)	2.0 (1.9)	2.2 (2.1)	1.5 (1.7)
0	54 (34%)	24 (31%)	19 (45%)
1	6 (4%)	2 (3%)	2 (5%)
2	51 (32%)	24 (31%)	9 (21%)
3	13 (8%)	8 (10%)	6 (14%)
4	18 (11%)	11 (14%)	4 (10%)
5	11 (7%)	6 (8%)	1 (2%)
6	2 (1%)	0 (0%)	1 (2%)
7	1 (1%)	1 (1%)	0 (0%)
8	1 (1%)	1 (1%)	0 (0%)
9	0 (0%)	0 (0%)	0 (0%)
10	1 (1%)	1 (1%)	0 (0%)
Care-needs level after a fracture			
Not required	23 (15%)	10 (13%)	0 (0%)
Lower level	89 (56%)	46 (59%)	24 (57%)
Higher level	46 (29%)	22 (28%)	18 (43%)
Usage of institutional care services after a fracture			
Yes	50 (32%)	23 (29%)	5 (12%)
No	108 (68%)	55 (71%)	37 (88%)
Duration of usage of long-term care (months) after a fracture^f^			
Mean (SD)	Not applicable	Not applicable	6.3 (4.2)
Median			5

^a^Included those who were admitted for a hip fracture between October 2012 and April 2013

^b^Included those who had not used long-term care before a fracture

^c^Not applicable, as some patients were admitted multiple times

^d^Some patients received different procedures

^e^Included comorbidity pre-hip fracture or in the month of an admission for a hip fracture

^f^Only relevant for the analysis of long-term care expenditure

**Fig. 2:**
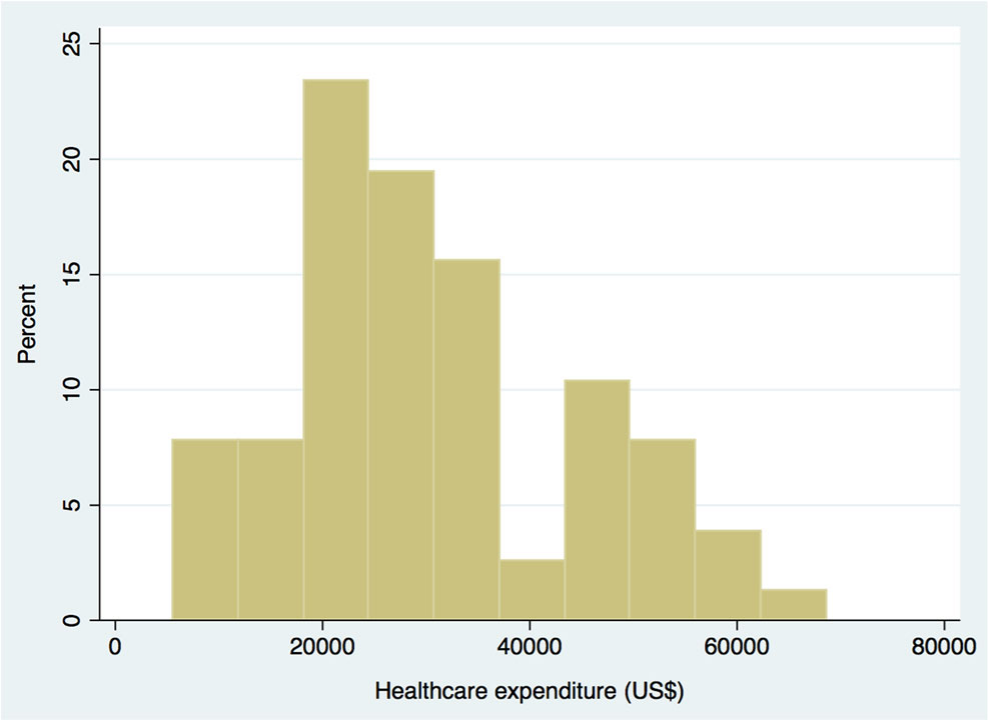
Distribution of healthcare expenditure post-hip fracture. One value was not included, in which pre-hip fracture expenditure exceeded post-hip fracture expenditure in the figure (*n* = 77)

For analyses of healthcare expenditure, we included only those admitted between October 2012 and April 2013 (*n* = 78) (Fig. 1). The mean and median total incremental payments including both inpatient and outpatient care during the period 6 months before and after the hip fractures were ¥2,600,000 (US$29,500) (95% CI ¥2,300,000–2,900,000, US$26,200–33,000) and ¥2,340,000 (US$26,600) (95% CI ¥2,150,000–2,840,000, US$24,400–32,300), respectively (Table 2). One individual in this cohort died during the hospitalization period after a 5-month stay at the hospital and, in this case, the expenditure for the sixth month was considered to be zero. The distribution of the healthcare expenditure is presented in Fig. 2. The mean incremental payments of inpatient and outpatient care were calculated to be ¥2,630,000 (US$29,800) and − ¥30,000 (− US$300), respectively. Out of 78 patients, 3 patients did not use outpatient care both pre- and post-hip fracture. The mean and median attributable payments were ¥2,320,000 (US$26,400) and ¥2,210,000 (US$25,200), respectively, and the proportion of mean attributable payments to the total mean incremental payments was 89%.

**Table 2:** Estimated healthcare and monthly long-term care expenditures (per person) post-hip fracture

	Mean	Median
Healthcare (*n* = 78)^a^	¥2,600,000 (2,300,000–2,900,000)^c^ US$29,500 (26,200–33,000)^c^	¥2,340,000 (2,150,000–2,840,000)^c^ US$26,600 (24,400–32,300)^c^
Monthly long-term care (*n* = 42)^b^	¥113,000 (85,000-141,000)^c^ US$1290 (970–1600)^c^	¥80,000 (54,000–114,000)^c^ US$910 (620–1300)^c^

¥88 was equivalent to US$1, which was the mean exchange rate from April 2012 to September 2013

^a^Calculated as the difference between the total payments 6 months before and 6 months after hip fractures

^b^Included those who had not used long-term care before a fracture and started long-term care after a fracture

^c^Bias-corrected confidence interval provided using bootstrapping

**Fig. 3:**
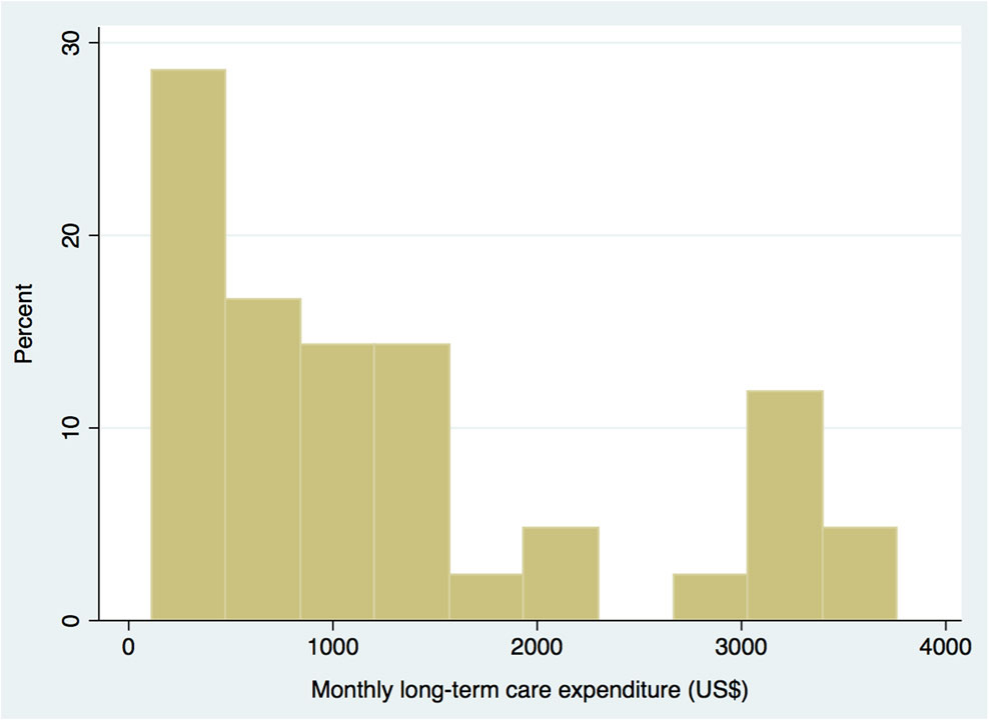
Distribution of monthly long-term care expenditure post-hip fracture (*n* = 42)

Among the 65 individuals without long-term care insurance prior to hip fracture, 42 individuals (65%) commenced long-term care insurance post-hip fracture. For analyses of long-term care expenditure, we included only those who started using it after a fracture (*n* = 42) (Fig. 1). The mean and median expenditures of monthly long-term care were ¥113,000 (US$1290) (95% CI ¥85,000–141,000, US$970–1600), and ¥80,000 (US$910) (95% CI ¥54,000–114,000, US$620–1300), respectively, with a mean duration of long-term care usage of 6.3 months (Table 2). Distribution of the long-term care expenditure is presented in Fig. 3. In a sub-analysis, in which we included those who started and those who did not start long-term care post-fracture (*n* = 65), the mean and median expenditures of monthly long-term care were ¥73,000 (US$830) (95% CI ¥52,000–96,000, US$590–1090), and ¥35,000 (US$400) (95% CI ¥21,000–66,000, US$230–750), respectively.

After we confirmed that assumptions were met, we performed multiple linear regressions to examine the associations of healthcare expenditure with various factors (*n* = 77). One individual had suffered from intracranial bleeding in the pre-hip fracture period, and subsequently had a higher expenditure in the pre-hip fracture period than in the post-fracture period. We excluded this individual in the regression analysis, because each expenditure was supposed to be a positive value. We found duration of hospital stay was positively associated with healthcare expenditure. An additional duration of hospital stay for 2 weeks was associated with a ¥22,000 (US$2450) higher healthcare expenditure (95% CI ¥1000–42,000, US$170–4740) (*p* = 0.036). We also found comorbidity to be negatively associated with healthcare expenditure (*p* = 0.015). Compared with a CCI score less than 4, a CCI score ≥ 4 was associated with lower healthcare expenditure by ¥796,000 (US$9000) (95% CI ¥157,000–1,434,000, US$1790–16,300) (Table 3). Of 77 patients with a hip fracture, 38 individuals were admitted to hospital A, 20 to hospital B, 6 to hospital C, and 5 to hospital D; the rest of the 8 individuals were admitted to 5 different hospitals (total of 10 hospitals). In the sensitivity analysis, we performed a mixed effect linear regression model, in which we treated the hospitals as random-effect variables and found the results were overall similar to those from the multiple linear regression and the negative association between the CCI score and healthcare expenditure persisted (*p* = 0.039) (linear regression test vs. linear model, *p* = 0.0723).

**Table 3:** The associations of expenditures with various factors among patients with a hip fracture

Variables	Healthcare^a^, US$ (*n* = 77)			Long-term care^b^, US$ (*n* = 42)		
	Adjusted *R*-squared 0.090			Adjusted *R*-squared 0.350		
	Unstandardized regression coefficient	*p* value	95% CI	Unstandardized regression coefficient	*p* value	95% CI
Birth year prior to or equal to 1929, equal to or after 1930 (ref)	− 3407	0.344	− 10,540, 3725	14	0.964	− 615, 644
Sex: women, men (ref)	220	0.956	− 7735, 8176	− 204	0.603	− 615, 644
Duration of hospital stay^c^	2454	0.036	168, 4740	Not applicable		
Procedures: hip replacement, other (ref)	886	0.803	− 6158, 7934	Not applicable		
Charlson Comorbidity Index ≥ 4, < 3 (ref)	− 9044	0.015	− 16,300, − 1788	258	0.541	−591, 1108
Care-needs level after a fracture^c^, lower level, higher level, no long-term care (ref)^d^	−3601	0.155	− 8602, 1400	696	0.022	108, 1285
Usage of institutional care services yes, no (ref)	Not applicable			1763	< 0.001	849, 2678

¥88 was equivalent to US$1, which was the mean exchange rate from April 2012 to September 2013

^a^Included those who admitted for a hip fracture between October 2012 and April 2013

^b^Included those who had not used long-term care before a hip fracture and started using it after a fracture

^c^Categorized as follows: duration (1) ≤ 2 weeks, (2) > 2 weeks, (3) > 4 weeks, (4) > 6 weeks, (5) > 8 weeks, (6) > 10 weeks

^d^Lower level (ref) and higher level only for long-term care analysis

In the multiple linear regressions to examine the associations of long-term care expenditure with various factors (*n* = 42), of which we also confirmed the assumptions were met, care-needs level after a fracture (*p* = 0.022), and usage of institutional care (*p* < 0.001) were associated with long-term care expenditure. An additional category of care-needs level requiring post-hip fracture was associated with an expenditure of ¥61,000 (US$700) (95% CI ¥10,000–113,000, US$110–1280); and usage of institutional care was associated with an expenditure of ¥155,000 (US$1800) (95% CI ¥75,000–236,000, US$850–2680) (Table 3).

## Discussion

Healthcare and monthly long-term care expenditures post-hip fracture were estimated to be ¥2,600,000 (US$29,500) and ¥111,300 (US$1290), respectively. We found associations of higher healthcare expenditure with duration of hospital stay and with lower baseline comorbidity, and associations of higher long-term care expenditure with higher care-levels needed and with usage of institutional care post-hip fracture.

Healthcare expenditure estimated in this study were a little higher than that of the previous study based on the results of three hospitals in Japan including one DPC (initial version of the DPC system) and two non-DPC hospitals, which presented ¥2,208,000 as the total hospitalization cost of hip fracture [3]. The mean age in that study was 82.3 years and the mean length of hospitalization was 43.7 days, which were both similar to those of our study. Their estimate was closer to our attributable difference in the payments made 6 months pre- and 6 months post-hip fracture, which was calculated only including claims representing the ICD 10 codes of S72 (i.e., hip fractures) (¥2,320,000 (US$26,400)). The authors estimated the post-hip fracture costs through reviewing the medical records, and the sum of the charges related to hip fractures presumably covered by healthcare insurance was calculated, which appears closer to our attributable difference than to our incremental difference. Our method followed that of a previous study, in which Kilgore et al. calculated the difference between healthcare expenditures 6 months before and after a fracture among Medicare beneficiaries in the USA [14]. One of the strengths of this approach was that an individual’s pre-hip fracture condition served as a control for the same individual’s post-fracture condition. We agree with Kilgore et al. that incremental differences in total payments are reasonable and reliable estimations associated with fractures, rather than the difference only in the attributable payments associated with fractures [14, 20].

The mean incremental payment of outpatient care in our study was calculated to be − ¥30,000 (− US$300). This is likely because the duration of stay is longer in Japan and outpatient care is not needed during hospitalization. The mean duration of stay in our study was 47.4 days, which was very similar to that of previous studies in Japan. The mean duration of stay was 43.7 days in Kondo et al.’s study involving three hospitals in Japan, and 45.9 days in Ishizaki et al.’s study (DPC/PDPS was not introduced in Japan at that time) [3, 16].

For estimating long-term care expenditure, we did not include those who had used long-term care insurance pre-hip fracture. A patient did not need to use the long-term care insurance during the hospitalization (with some exceptions such as home safety equipment after discharge), making long-term care expenditure potentially lower for those who had already used the long-term care insurance for other reasons prior to a hip fracture. We therefore included those who had not used long-term care insurance pre-hip fracture and commenced long-term care insurance post-hip fracture, as long-term care expenditure pre-hip fracture was 0 for those individuals. However, we might have underestimated the amount of long-term care expenditure, as our estimate was likely to have been based on a less frail population (i.e., those who did not use long-term care insurance prior to their hip fracture). Indeed, of 65 individuals who had not used long-term care prior to hip fracture, only 5 individuals (8%) started to use facility care post-hip fracture. We must also address the fact that various patient post-fracture follow-up periods were permitted, and some patients may have had only a very short observational period with the mean observation period of long-term care usage of 6.3 months in this study. Further study with a longer observation period (i.e., until death) is needed for better estimation of long-term care expenditure.

Healthcare expenditure was negatively associated with CCI scores in this study. Because this association was inconsistent with our hypothesis and was unexpected, we explored possible explanations for the association extensively. First, we performed univariate regression analysis and found a negative association (*p* = 0.014). Next, we adapted several different categorizations of CCI scores based on previous studies and the distribution of the CCI scores in this study [21, 22] and found negative associations between higher levels of comorbidity and lower amounts of healthcare expenditure persisted including, but not limited to the following: (a) < 6 or ≥ 6; (b) 0, 1–2, or ≥ 3; and (c) 0, 1–3, or ≥ 4 (*p* values of 0.034, 0.035, and 0.009, respectively). Third, we used the original CCI definition (< 5 or ≥ 5) instead of the updated and reweighted version in 2011, but the association remained (*p* = 0.013). Fourth, we performed a multiple linear regression including each component of CCI instead of the total score of CCI and found no single component to be significantly associated with the expenditure. Fifth, we examined the association of the duration of stay with CCI scores, adjusted for the same set of covariates (age, sex, functional status, and procedure), and found that CCI was not associated with duration of stay (*p* = 0.884). Sixth, we ran analyses in which we only removed duration of stay as one of the covariates from the original linear regression, as the duration of stay could function as an intermediate variable between higher CCI and lower expenditure, but the negative association between higher CCI and lower expenditure remained (*p* = 0.020). Seventh, we also ran analyses in which we only removed care-needs levels, but again the association persisted (*p* = 0.010). Eighth, we excluded three outliers, of which the studentized residuals exceeded + 2 or − 2, and found that the association persisted (*n* = 74, *p* = 0.005). Ninth, we also excluded one individual with closed reduction or wiring only, and found that the association remained (*n* = 76, *p* = 0.012). Tenth, we explored the possible transformations to address the skewed dependent variable, healthcare expenditure, although we did not consider that we violated the assumption of the normality of the residual. The results, however, with the square root-transformation and the log-transformation, which emerged as the best and second-best transformations, still showed a significant association between higher CCI scores and lower expenditure (*p* = 0.016 and 0.022, respectively). Furthermore, we ran a generalized linear model regression with log link and gamma distribution and found that the negative association persisted (*p* = 0.010). Finally, we estimated the sum of healthcare and long-term care expenditures post-hip fracture, in which long-term care expenditure was calculated as (i) the difference in 6 months pre- and 6 months post-hip fracture and (ii) 6 months post-hip fracture only. We then examined the association between the sum of the expenditures and CCI scores adjusted for the same set of covariates and found that the sum of the expenditures was no longer associated with CCI scores, regardless of the calculations of long-term care expenditure post-hip fracture (i.e., (i) *p* = 0.506; (ii) *p* = 0.486).

The finding between the sum of the expenditures and CCI scores suggested that those who had higher CCI scores might tend to receive post-hip fracture care through long-term care insurance, as some long-term care facilities could provide medical care and rehabilitation to some extent. On the other hand, those who had lower CCI scores might be better candidates for acute rehabilitation facilities, which are covered through healthcare insurance. This finding may therefore further emphasize the importance of considering post-hip fracture care as a combination of healthcare and long-term care. The association between the CCI scores and the duration of hospital stay may also explain our findings. In our study, duration of hospital stay was positively associated with healthcare expenditure, as we hypothesized, which is consistent with previous studies from the UK [23, 24]. On the other hand, CCI scores were not associated with duration of stay (mean 47.4 days) in our study. In a previous US study, in which mean duration of stay was 5.84 days, CCI scores were associated with prolonged duration of stay, leading to increased hospital costs post-hip fracture [21]. In another previous study in the USA, comorbidities were associated with length of stay (mean approximately 6 days) and cost of hospitalizations [17]. Non-DPC/PDPS hospitals in Japan often function to provide sub-acute or chronic care, which may be the reason CCI scores were not associated with duration of stay. In addition, there might be unmeasured confounding factors that may have affected the association in our study. For instance, the relationships between higher level of the American Society of Anesthesiologists (ASA) and higher hospital costs of hip fracture were presented in previous studies [18, 25]. Another possible explanation of the association between higher CCI scores and lower healthcare expenditure was that CCI scores based on healthcare claims data in Japan may not be accurate. This is because the suspected diagnoses are often placed in the claims data so that examinations or medications can be covered by the fee-for-services system, although conditions with a “suspicious” flag, which suggested that the diagnoses were placed to justify diagnostic procedures, were not included to obtain CCI scores in this study. Further studies are needed to examine the associations of comorbidity with healthcare and long-term care expenditures post-hip fracture.

Monthly long-term care expenditure was positively associated with higher care-levels needed after a fracture and usage of institutional care, which was consistent with our hypotheses. Each care-level determined the maximum amount of long-term care services [6]. A previous study in Japan showed long-term care insurance expenditure was associated with institutional care usage, consistent with our study findings [26].

We noted several limitations. Firstly, the observational period of 1.5 years was not enough to capture lifelong data about long-term care post-hip fracture as discussed above. Second, we chose an approach to compare the differences in the payments for 6 months pre- and post-hip fracture, which reduced the number of fracture cases used for this study. Third, we did not include DPC/PDPS hospitals, as data from DPC/PDPS hospitals was seemingly incomprehensive at the time of this analysis. There was a possibility that patients with hip fracture were more often admitted to DPC/PDPS hospitals. However, a previous study in Japan suggested that total hospitalization costs after hip fractures might not differ between DPC/PDPS and non-DPC/PDPS hospitals, as DPC/PDPS hospitals might reduce duration of stay at these hospitals, but subsequently increase duration of stay at sub-acute/rehabilitation hospitals in Japan [3]. We also did not include those receiving public assistance.

On the contrary, our study has several strengths. This is a population-based study and the first study to estimate healthcare and long-term care expenditures post-hip fracture by using claims data in Japanese facilities. Our data were derived from a population greater than 400,000 in 2012 (approximately 21% were aged ≥ 65 years as of October 2012). In October 2012, 24.1% of the population in Japan were aged ≥ 65 years [27], with rural areas having more older populations and urban and surrounding areas having fewer older populations. Therefore, the City of Kashiwa, which we obtained data from was considered to be a typical suburb of a large city (i.e., Tokyo) in terms of the ratio of the older population. We took a unique approach, in which we merged healthcare claims data into long-term care claims data at a depersonalized individual level. This method enabled us to add functional status at the time of discharge to healthcare claims data, to add diagnoses of hip fractures and comorbidities to long-term care claims data, and to calculate healthcare and subsequent long-term care expenditures post-hip fracture. Finally, the results of this study can be used for further cost-effectiveness analyses, which would allow more reliable estimates for costs associated with hip fractures [28]. The importance of cost-effectiveness analyses has increased because in 2016 the Japanese Ministry of Health, Labour and Welfare started to use the results of cost-effectiveness analyses to update the medical fee schedule every 2 years for expensive treatments that the healthcare insurance system in Japan covers [29].

In conclusion, healthcare and monthly long-term care expenditures post-hip fracture were estimated to be ¥2,600,000 ($29,500) and ¥113,000 (US$1290), respectively. This is the first study to estimate healthcare and long-term care expenditures post-hip fracture using claims data in Japanese facilities. Further studies are needed for a longer observation period to capture the lifelong course of long-term care post-hip fracture (i.e., until death) at both DPC/PDPS and non-DPC/PDPS hospitals.
