# Correction to: Estimated expenditures for hip fractures using merged healthcare insurance data for individuals aged ≥ 75 years and long-term care insurance claims data in Japan

**DOI:** 10.1007/s11657-019-0562-9

**Published:** 2019-02-27

**Authors:** Takahiro Mori, Nanako Tamiya, Xueying Jin, Boyoung Jeon, Satoru Yoshie, Katsuya Iijima, Tatsuro Ishizaki

**Affiliations:** 1 Research and Development Center for Health Services, Faculty of Medicine, University of Tsukuba, 1-1-1 Tenno-dai, Tsukuba, Ibaraki 305-8575 Japan; 2 Department of General Internal Medicine, Eastern Chiba Medical Center, Togane, Japan; 3 Department of General Medical Science, Graduate School of Medicine, Chiba University, Chiba, Japan; 4 Institute of Gerontology, University of Tokyo, Tokyo, Japan; 5 Human Care Research Team, Tokyo Metropolitan Institute of Gerontology, Tokyo, Japan

**Correction to: Archives of Osteoporosis**

10.1007/s11657-018-0448-2

The article *Estimated expenditures for hip fractures using merged healthcare insurance data for individuals aged ≥ 75 years and long-term care insurance claims data in Japan*, written by Takahiro Mori, Nanako Tamiya, Xueying Jin, Boyoung Jeon, Satoru Yoshie, Katsuya Iijima, and Tatsuro Ishizaki was originally published electronically on the publisher’s internet portal (currently SpringerLink) on 30 March 2018 without open access.

With the author(s)’ decision to opt for Open Choice the copyright of the article changed on 30 January 2019 to © The Author(s) 2019 and the article is forthwith distributed under the terms of the Creative Commons Attribution 4.0 International License (http://creativecommons.org/licenses/by/4.0/), which permits use, duplication, adaptation, distribution, and reproduction in any medium or format, as long as you give appropriate credit to the original author(s) and the source, provide a link to the Creative Commons license and indicate if changes were made. The original article has been corrected.

